# Identification of *bla*_OXA-23_ gene in resistant *Pseudomonas aeruginosa* strains isolated from cows and humans in Basra province, Iraq

**DOI:** 10.14202/vetworld.2024.1629-1636

**Published:** 2024-07-26

**Authors:** Alyaa Sabti Jasim, Abeer Laily Mohammed, Wameedh Hashim Abbas, Hanaa Khaleel Ibraheim, Hasanain A.J. Gharban

**Affiliations:** 1 Department of Microbiology, College of Veterinary Medicine, University of Basrah, Basra, Iraq; 2 Department of Microbiology, Al-Zahraa College of Medicine, University of Basrah, Basra, Iraq; 3 Department of Internal and Preventive Veterinary Medicine, College of Veterinary Medicine, University of Wasit, Wasit, Iraq

**Keywords:** antibiotic susceptibility test, biofilm formation, *bla_Oxacillinases-23_*, conventional polymerase chain reaction, cow milk, nasal discharge, sputum, urine

## Abstract

**Background and Aim:**

*Pseudomonas aeruginosa* is an infectious agent of great importance for animals and humans. It causes serious infections that show high resistance to antibiotics. This study investigated the molecular detection of *bla_OXA-23_* gene in antibiotic-resistant *P. aeruginosa* strains isolated from cows and humans.

**Materials and Methods:**

In total, 120 samples, comprised 60 from cows (30 milk and 30 nasal discharge) and 60 from their owners (30 urine and 30 sputum), were individually collected, cultured, and tested for *P. aeruginosa* through molecular analysis targeting the *bla_OXA-23_* gene. *P. aeruginosa* antibiotic-resistant isolates were identified by performing antibiotic susceptibility testing and detecting biofilm formation.

**Results:**

In total, 74.17% positive *P. aeruginosa* isolates, including 66.67% and 81.67% for cows and humans, respectively. Subsequently, positive cow isolates were detected in 60% of milk samples and 73.33% of nasal discharge samples; while positive human isolates were detected in 76.67% of urine samples and 86.66% of sputum samples. Targeting *bla_OXA-23_* gene, 58.43% of cultured isolates were positive for *P. aeruginosa* by polymerase chain reaction. Respectively, positive isolates were detected in 66.67% and 45.46% of cow milk and nasal discharges as well as in 60.87% and 61.54% of human urine and sputum. The antibiotic susceptibility test revealed that all isolates were resistant to all applied antibiotics, particularly imipenem. Results of biofilm formation revealed 67.31% total positives, including 51.43% strong, 34.285% moderate, and 14.285% weak reactions. In addition, although values of the total positive cows and humans differed insignificantly, total positives showed insignificant variation between values of milk and nasal discharges of cows as well as between urine and sputum of humans; however, significant differences were identified in the distribution of strong, moderate, and weak positivity of these samples.

**Conclusion:**

Antibiotic overuse contributes extensively to increasing the prevalence of resistant *P. aeruginosa* isolates carrying the *bla_OXA-23_* gene in both cows and humans. Furthermore, studies in other Iraqi areas are necessary to support our findings. The main limitations include that the number of tested samples is relatively low, and there is a need to use a large number of samples from different sources. Also, the current methods for detection of resistant isolates are still culture-based approaches.

## Introduction

Among the pathogenic microorganisms that cause human infections, *Pseudomonas aeruginosa* remains one of the most common agents of outbreaks in hospitals worldwide [1]. This pathogen is the most common species in the *Pseudomonas* genus, comprising 144 species, 25% of which are associated with human illnesses [2]. This bacterium can infect the respiratory, urinary, and gastrointestinal systems, skin, bones, soft-tissues, blood, and eyes, causing a wide range of systemic illnesses, particularly in patients with serious burns and immunodeficiency [3, 4].

The pathogenic arsenal of *P. aeruginosa* includes various virulence factors that neutralize the host’s defenses, induce tissue damage, and form biofilms, all of which boost the microorganism’s competitiveness [5, 6]. Other major virulence factors, such as fimbriae, flagella, pili-type, and superficial polysaccharides IV, play a role in the colonization of bacteria [7]. Furthermore, biofilm formation is a fundamental and crucial virulence component that enhances bacterial survival under adverse conditions, such as the presence of antiseptics or dryness [8]. Biofilms are also one of the primary antibiotic resistance methods, facilitating the horizontal transfer of genes among sensitive and antibiotic-resistant bacteria [9]. Biofilm also hinders bacteria, drugs, and immunological responses [10]. Given their broad antibacterial range, carbapenems are reliable and efficient antibiotics against many pathogenic organisms. They are used to treat severe nosocomial infections caused by cephalosporin-resistant bacteria [11].

The *bla_OXA-23_* gene is a member of the Class D carbapenemase gene group, which is considered the first group of OXA-type B-lactamases capable of hydrate carbapenems and broad-spectrum cephalosporins [12]. The *bla_OXA-23_* gene may be found on a chromosome in addition to plasmids and has been linked to mobile genetic components. This group was deemed the first group of OXA-type B-lactamases *bla_OXA-23_* that cause carbapenem resistance, and this has been documented globally [13]. These microorganisms are frequently linked to multidrug resistance and extended drug resistance. Thus, many infections caused by *P. aeruginosa* are difficult to treat, resulting in mortality, morbidity, and an enormous financial strain on individuals [14]. Strains of *P. aeruginosa* are among the most common pathogenic bacteria that produce extended-spectrum beta-lactamases [15]. Thus, therapeutic trials on infections caused by *P. aeruginosa* carrying *bla_OXA-23_* are challenging.

In Iraq, although resistant *P. aeruginosa* strains *bla_OXA-23_* gene have been investigated in Al-Nasseryia [16], Al-Najaf [17, 18], Baghdad [19], and Al Muthanna [20], no studies have been carried out in Basra province. Therefore, this study aimed to detect the *bla_OXA-23_* gene in antibiotic-resistant *P. aeruginosa* strains isolated from cows and humans.

## Materials and Methods

### Ethical approval

This study was approved by the Scientific Committees of the College of Veterinary Medicine and Al-Zahraa College of Medicine (University of Basrah), and the College of Veterinary Medicine (University of Wasit) (No.447/CVM-UW/10-4-2022).

### Study period and location

The study was conducted from February to October 2023. The samples were processed at Microbiology Laboratory, College of Veterinary Medicine, University of Basrah.

### Sample collection

In total, 120 samples; 60 from cows (30 milk and 30 nasal discharge) and 60 from their owners (30 urine and 30 sputum), were collected randomly as described by previous studies [20, 21]. All samples were collected under aseptic conditions from different areas of Basra province, Iraq, and transported in a cool box (4°C) to the Microbiology Laboratory, College of Veterinary Medicine, University of Basrah.

### Identification of bacterial isolates

The samples were initially cultured in MacConkey and blood agars (HiMedia, India) and incubated at 37°C for 24 h. The suspected colonies were re-cultured based on their morphological characteristics to obtain the purified colonies. For additional confirmation, biochemical tests were performed as described by Al-Janahi [21].

### Molecular detection of isolates

DNA was extracted from the purified isolates according to the manufacturer’s instructions using the Genomic DNA Mini Kit (Geneaid, Taiwan). Targeting the *bla_OXA-23_* gene, one set of primers was used (F[5´-TGGAAGGGCGAGAAAAGGTC-3´] and R[5´-TTGCCCAACCAGTCTTTCCA-3´]) to prepare MasterMix tubes (Promega, USA) at 25 μL (5 μL DNA template, 2 μL forward primer, 2 μL reveres primer, 16 μL PCR water) final volume [22]. For the amplification reaction, the polymerase chain reaction (PCR) tubes were transferred to a thermal cycler and subjected to the following conditions: One cycle for initial denaturation (94°C for 3 min); 35 cycles for denaturation (95°C for 25 s), annealing (52°C for 45 s), extension (72°C for 50 s); and 1 cycle for final extension (72°C for 5 min). Electrophoresis of agarose gel (1.5%) stained with ethidium bromide (Biotech, Canada) at 80 Am and 100 Volt for 90 min was performed; then, positive samples were observed using an ultraviolet transilluminator (Clinx Science, China) at approximately 400 bp.

### Antibiotic susceptibility testing

Following the guidelines of the Clinical Laboratory Standards Institute (CLSI) [23], the test was performed using the disc diffusion method to determine the resistance pattern. Briefly, bacterial suspensions were overnight cultured on Muller-Hinton agar (MHA; Oxoid, Hampshire, England), and the suspension density was modified to 0.5 McFarland standard, equivalent to roughly 1.5 × 10^8^ colony forming unit/mL. The surface of the MHA (HiMedia) plate was coated with a solution containing sterile cotton swabs. After adding the disks with antibiotics, the dishes were airdried and incubated overnight at 37°C. The sizes of the inhibition zones around the disks were determined. The results were interpreted according to the CLSI [23].

### Biofilm formation

Isolates of *P. aeruginosa* were inoculated into 5 mL of trypticase soy broth (TSB) and incubated at 37°C overnight for biofilm development. After preparing a McFarland standard concentration of 0.5 in TSB, 100 μL of the dilution was added to each well of a flat-bottomed polystyrene 96-well microtiter plate. After 24 h of incubation at 37°C, the supernatant was harvested, and the wells were washed with 0.9% NaCl solution. Then, identical biofilms were treated with 99% ethanol, and the surface of each plate was dried in air and stained with 1.5% crystal violet for 20 min. Finally, the dye was solubilized in 150 μL of 30% (v/v) acetic acid, and the absorbance was measured at 450 nm using an enzyme-linked immunosorbent assay reader (BioTek, USA) [24].

### Statistical analysis

The obtained data were documented and tabled in Microsoft Office Excel Software version 2016 (Microsoft, Washington, USA); while statistically, one pair *t*-test and one-way analysis of variance in GraphPad Prism Software version 6.0.1 (GraphPad Software, Inc, USA) were used to estimate significant differences between study groups at p < 0.05 [25].

## Results

**Figure-1:**
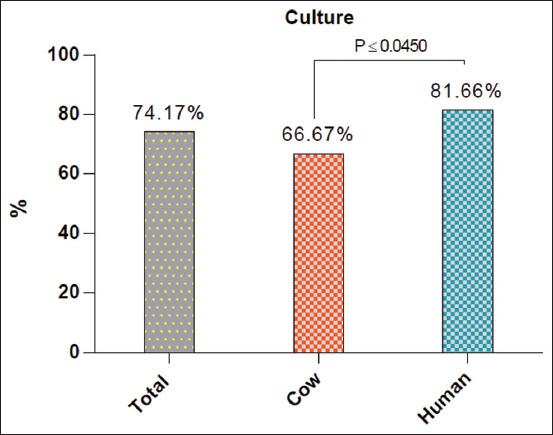
Total positive *Pseudomonas aeruginosa* isolates by culture in the study population, cows and humans.

Among the cultured samples, 74.17% (89/120) positive isolates, including 66.67% (40/60) and 81.67% (49/60), were positive for humans (Figure-1). According to sample type, the positive isolates from cows were identified in 60% (18/30) of milk and 73.33% (22/30) of nasal discharges, while in humans, positive results were detected in 76.67% (23/30) and 86.67% (28/30) of urine and sputum samples, respectively (Table-1).

**Table-1:** Total results of positive *P. aeruginosa* in collected specimens of study populations (cows and humans) using the culture and biochemical tests.

Species	Specimen	Total no.	Positive		p-value

			No.	%	
Cow	Milk	30	18	60	0.0466
	Nasal discharge	30	22	73.33*	
	Total	60	40	66.67	-
Human	Urine	30	23	76.67	0.0394
	Sputum	30	26	86.66*	
	Total	60	49	81.66	-

*P. aeruginosa*=*Pseudomonas aeruginosa.* (*) Refer to a significant increase in vertically compared values at p < 0.05. The first p-value (0.0466) refers to significant differences between the results of milk and nasal discharge of cow samples. The second p-value (0.0394) refers to significant differences between values of urine and sputum of human samples

**Figure-2:**
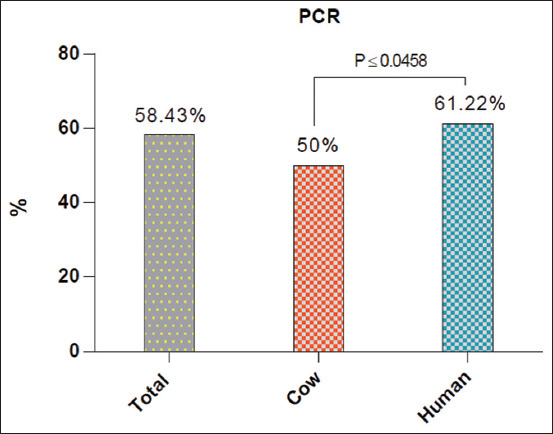
Total positive *Pseudomonas aeruginosa* isolates by polymerase chain reaction in the study population, cows and humans.

**Figure-3:**
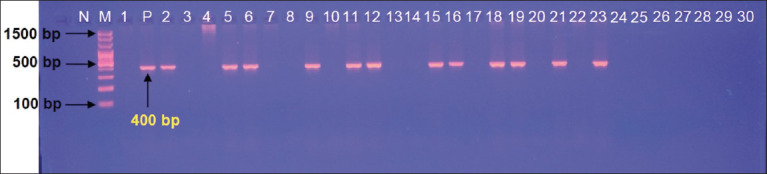
Agarose-gel electrophoresis of polymerase chain reaction products at 80 Am and 100 Volt; Line (M): Ladder marker; Line (N): Negative control; Line (P): Positive control; Lines (1, 3, 4, 7, 8, 10, 13, 14, 17, 20, 22, and 24–30): Representative of negative samples; Lines (2, 5, 6, 9, 11, 12, 15, 16, 18, 19, 21, and 23): Representative of positive *Pseudomonas aeruginosa* strains to *bla_OXA-23_* gene at approximately 400 bp product size in the study population, cows and humans.

Targeting the *bla_OXA-23_* gene, the PCR findings confirmed that 58.43% (52/89) of the cultured isolates were positive for *P. aeruginosa* (Figures-2 and 3). According to the type of samples, 66.67% (12/18) and 45.46% (10/22) of milk and nasal discharges of cow isolates were positive, whereas 60.87% (14/23) of urine and 61.54% (16/26) of sputum human isolates were positive (Table-2).

**Table-2:** Total positive *P. aeruginosa* isolates targeting the *bla*_OXA-23_ genein cow and human specimens using the PCR assay.

Species	Specimen	Total no.	Positive		p-value

			No.	%	
Cow	Milk	18	12	66.67*	0.0399
	Nasal discharge	22	10	45.46	
	Total	40	22	55	-
Human	Urine	23	14	60.87	0.075
	Sputum	26	16	61.54	
	Total	49	30	61.22	-

*P. aeruginosa*=*Pseudomonas aeruginosa*, PCR=Polymerase chain reaction. (*) Refer to non significant variation between vertically compared values (p > 0.05).

In this study, antibiotic susceptibility testing of molecularly positive *P. aeruginosa* isolates to *bla_OXA-23_*gene recorded that all isolates were resistant to all applied antibiotics (Table-3). However, a higher level of significant resistance was observed to imipenem (96.15%) and lowered to ceftazidime (57.69%) compared with other antibiotics: meropenem (86.53%), gentamycin (84.61%), piperacillin (76.92%), cefotaxime (75%), and ciprofloxacin (71.15%).

**Table-3:** Results of antibiotic susceptibility test among positive *P. aeruginosa* strains by PCR.

Results	Antibiotic			p-value

	Resistant	Intermediate	Sensitive	
Imipenem (10 μg)	50 (96.15%)*	0 (0%)	2 (3.84%)	0.0019
Meropenem (10 μg)	45 (86.53%)*	3 (5.76%)	4 (7.69%)	0.0026
Cefotaxime (30 μg)	39 (75%)*	4 (7.69%)	9 (17.30%)	0.0084
Ceftazidime (30 μg)	30 (57.69%)*	7 (13.46%)	15 (28.84%)	0.013
Piperacillin (100 μg)	40 (76.92%)*	2 (3.84%)	10 (19.23%)	0.0082
Ciprofloxacin (5 μg)	37 (71.15%)*	2 (3.48%)	13 (25%)	0.0086
Gentamycin (10 μg)	44 (84.61%)*	3 (5.76%)	5 (9.61%)	0.0077
p-value	0.0156	0.0313	0.0156	-

*P. aeruginosa*=*Pseudomonas aeruginosa*, PCR=Polymerase chain reaction. (*) Refers to significant increase in horizontally compared values at p < 0.05.

### Biofilm formation

**Figure-4:**
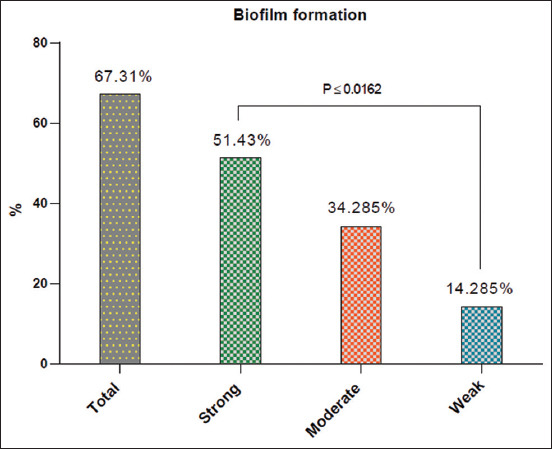
Total results and degree of positivity of biofilm formation test in positive *Pseudomonas aeruginosa* strains in the study population, cows and humans.

**Figure-5:**
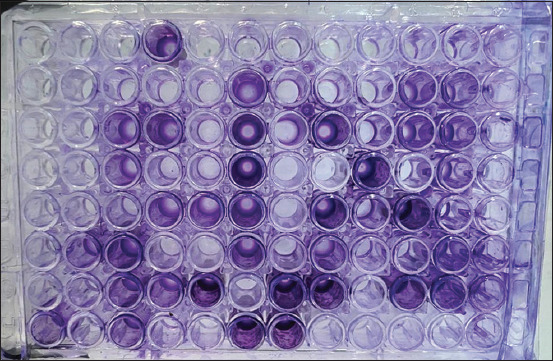
Microplate for testing *Pseudomonas aeruginosa* isolates by the biofilm formation test.

Examination of all molecularly positive *P. aeruginosa* isolates for biofilm formation revealed a total of 67.31% (35/52) positive samples, including 51.43% (18/35), 34.285% (12/35), and 14.285% (5/35) for strong, moderate, and weak reactions, respectively (Figures-4 and 5). In addition, insignificant variation (p < 0.072) was observed between the total positivity of cows [68.18% (15/22)] and humans [66.67% (20/30)]. However, cow isolates showed a significant elevation (p < 0.0479) in strong positivity [(55% (11/20)] and a significant reduction in moderate positivity [30% (6/30)] when compared with humans [46.67% (7/15) and 40% (6/15)], but not in weak positivity; in which, no significant variation (p < 0.0607) was observed between cows [13.33% (2/15)] and humans [15% (3/20)] positive isolates (Table-4).

**Table-4:** Total results of biofilm formation positive *P. aeruginosa* strains.

Sample	Total no.	Total no. of positive	Degree of biofilm formation			p-value

			Strong	Moderate	Weak	
Cow	22	15 (68.18%)	7 (46.67%)	6 (40%)	2 (13.33%)	0.0145
Human	30	20 (66.67%)	11 (55%)	6 (30%)	3 (15%)	0.0199
p-value		0.072	0.0479	0.043	0.0607	-

*P. aeruginosa*=*Pseudomonas aeruginosa*

Concerning cows, no significant variation (p < 0.0653) was observed between the total positive samples of milk [66.67% (8/12)] and nasal discharge [70% (7/10)]; however, strong and moderate positivity was significantly increased in milk [50% (4/8) and 25% (2/8), respectively] compared with nasal discharge [42.85% (3/7) and 0% (0/7), respectively], but moderate positivity was higher in nasal discharge [57.14% (4/7)] than milk [25% (2/8)], (Table-5).

**Table-5:** Results of biofilm formation among cows’ positive *P. aeruginosa* strains.

Sample	Total no.	Total no. of positive	Degree of positivity			p-value

			Strong	Moderate	Weak	
Milk	12	8 (66.67%)	4 (50%)*	2 (25%)	2 (25%)*	0.0415
Nasal discharge	10	7 (70%)	3 (42.85%)	4 (57.14%)*	0 (0%)	0.0089
p-value		0.0653	0.049	0.0368	0.0265	-

*P. aeruginosa*=*Pseudomonas aeruginosa.* (*) Refers to significant increase in horizontally compared values at p < 0.05.

Regarding human isolates, the total positivity of urine [64.26% (9/14)] and sputum [68.75% (11/16)] as well as the strong degree of positivity of both samples [55.55% (5/9) and 54.54% (6/11), respectively] differed insignificantly (p > 0.05). However, significant elevations in moderate and weak reactivity values were observed in urine [33.33% (3/9) and 11.11% (1/9), respectively] compared with sputum [27.27% (3/11) and 18.18% (2/11), respectively], (Table-6).

**Table-6:** Results of biofilm formation among human positive *P. aeruginosa* strains.

Sample	Total no.	Total no. of positive	Degree of positivity			p-value

			Strong	Moderate	Weak	
Urine	14	9 (64.29%)	5 (55.55%)	3 (33.33%)*	1 (11.11%)	0.0433
Sputum	16	11 (68.75%)	6 (54.54%)	3 (27.27%)	2 (18.18%)*	0.0133
p-value		0.0693	0.0959	0.0454	0.0449	-

*P. aeruginosa*=*Pseudomonas aeruginosa.* (*) Refers to significant increase in horizontally compared values at p < 0.05.

## Discussion

Antibiotic-resistant bacteria are difficult to treat with standard antibiotics because of their typical biofilm development and the presence of many pathogenic genes. Phenotypic and biochemical approaches are widely used to identify *P. aeruginosa* and remain the standard and reliable routine techniques [26]. Molecular biology methods can identify *P. aeruginosa* more accurately than traditional phenotypic or biochemical techniques [27]. This bacterium boasts a remarkable genotypic diversity due to its adaptability to various surroundings, expressing rare phenotypic characteristics [28]. Several researchers have studied various areas in many countries using molecular techniques as highly sensitive and specific tools for diagnosing *P. aeruginosa* [29–31].

In comparison with other studies, Rouhi and Ramazanzadeh [32] reported that 91.78% of isolates were *P. aeruginosa* with presence *bla_OXA-23_* gene in 11.19% of the strains; while Tarafdar *et al*. [33] found that *bla_OXA-23_* gene was detected in 70.83% of *P. aeruginosa* isolates. Human respiratory system isolates had much higher levels of *bla_OXA-23_* than animal isolates [34]. Hence, it was hypothesized that pathogenic genes of *P. aeruginosa* could exhibit varying levels of intrinsic virulence and pathogenicity [35]. Gonçalves *et al*. [36] detected *bla_OXA-23_* in 87.5% of carbapeneme-resistant *P. aeruginosa* isolates.

The study supports the notion that the *bla_OXA-23_* gene is extensively present in antibiotic-resistant *P. aeruginosa* isolates and may enhance the strains’ virulence, in accordance with earlier speculation [37]. The significance of natural microbiota as a reservoir for multidrug-resistant microbes in humans and animals is often overlooked. The antimicrobial resistance and virulence of *P. aeruginosa* can lead to increased morbidity and mortality in infected patients [38–40]. Imipenem resistance was confirmed in all *P. aeruginosa* isolates. Our study yielded results consistent with the existing literature [41–43]. Metallo-beta-lactamase (class D MBLs) and carbapenem-hydrolyzing oxacillinase are the primary sources of carbapenem resistance [44]. However, the rates of resistance to antibiotics in research vary depending on factors such as antibiotic type, genetic variance in bacteria and strains, and the variability in antibiotic usage habits among different countries [45]. Overuse of antibiotics and the development of antibiotic resistance genes may lead to resistance strains because *P. aeruginosa* is well known for its high inherent and acquired susceptibility to a wide spectrum of antibiotics [46]. Antimicrobial resistance is a public health concern because it alters the natural bacterial community and increases resistance levels [47].

*P. aeruginosa* resistance can be acquired through horizontal transfer of genes or mutations because adaptive immunity involves biofilm development, which serves as a diffusion obstacle, limiting antibiotic access to the bacterial cell [48]. Biofilm formation enhances antibiotic resistance and pathogenicity, resulting in chronic infections [49]. All resistant isolates in the study were capable of forming diverse biofilms. Antibiotic resistance and biofilm development in *P. aeruginosa* have been shown to have a strong correlation [50–52]. The presence of antibiotic-resistant *P. aeruginosa* isolates with high biofilm generation rates further substantiates our research findings.

## Conclusion

Antibiotic overuse extensively increases the prevalence of resistant *P. aeruginosa* isolates carrying the *bla_OXA-23_* gene and variable degrees of biofilm formation in both cows and humans. Transmission of resistant isolates to humans can occur both directly through milk consumption and indirectly through contaminated fomites. The *bla_OXA-23_* gene presence in *P. aeruginosa* isolates varied considerably between clinical samples of humans and cows. To strengthen our results, investigation in other Iraqi areas is essential. The main limitation of the present study includes the relatively low number of tested samples and the need to use a large number of samples from different sources. Also, the current methods for detecting resistant isolates are still culture-based approaches.

## Authors’ Contributions

ASJ and HKI: Collection of cow samples, isolation of *P. aeruginosa* isolates, and antibiotic susceptibility testing. ALM and WHA: Collection of human samples and molecular testing. HAJG: Biofilm testing with collection and statistical analysis of obtained data. All authors have read, reviewed, and approved the final manuscript.
